# Systematic review of the predictive effect of MSI status in colorectal cancer patients undergoing 5FU-based chemotherapy

**DOI:** 10.1186/s12885-015-1093-4

**Published:** 2015-03-21

**Authors:** Elizabeth M Webber, Tia L Kauffman, Elizabeth O’Connor, Katrina AB Goddard

**Affiliations:** Center for Health Research - Kaiser Permanente Northwest, 3800 N Interstate Avenue, Portland, OR 97227 USA

**Keywords:** Genetic, Pharmacogenomic, Meta analysis, Lynch syndrome

## Abstract

**Background:**

We systematically reviewed the evidence for the interaction of microsatellite instability status (MSI) and treatment with 5FU in colorectal cancer to determine how well MSI status predicts health outcomes in patients undergoing 5FU-based chemotherapy.

**Methods:**

We conducted a search of four electronic databases through June 2013. We considered studies that included both colorectal cancer patients treated with 5FU-based chemotherapy and untreated patients with survival outcomes presented by MSI status.

**Results:**

We identified 16 studies for qualitative analysis (9,212 patients) with 14 studies eligible for meta-analysis. The microsatellite stable (MSS) group showed an effect of 5FU treatment on disease-free survival (HR of 0.62 [95% CI: 0.54, 0.71]) and overall survival (HR of 0.65 [95% CI: 0.54, 0.79]), indicating that MSS patients who received 5FU treatment had longer survival than MSS patients who were untreated. The effect of 5FU treatment was not statistically significant for microsatellite high (MSI-H) patients for disease-free survival (HR of 0.84 [95% CI: 0.53, 1.32]) or overall survival (HR 0.66 [95% CI: 0.43, 1.03]). However, the summarized point estimates of the effects of 5FU treatment for the MSS and MSI-H groups were not different at a statistically significant level.

**Conclusions:**

Our analyses indicate that treatment with 5FU-based chemotherapy improves disease-free and overall survival in CRC patients, but that there is no difference in the effect of treatment based on MSI status. Therefore, the use of MSI status to guide treatment decisions about the use of 5FU treatment for CRC has no significant benefits for patients.

**Electronic supplementary material:**

The online version of this article (doi:10.1186/s12885-015-1093-4) contains supplementary material, which is available to authorized users.

## Background

Colorectal cancer (CRC) is the third most commonly diagnosed cancer in the United States; about 1 in 20 Americans will develop CRC in their lifetime. Although the death rate from CRC has been dropping, it remains the third leading cause of cancer-related deaths [1]. About 15% of CRC tumors develop via a pathway characterized by defective function of the DNA mismatch repair (MMR) system. MMR deficiency most commonly occurs through epigenetic inactivation of the *MLH1* gene in sporadic CRCs, but can also occur through inherited mutations in any one of four genes (i.e., *MLH1*, *MSH2*, *MSH6*, and *PMS2*). Tumors with MMR deficiency exhibit a high frequency of microsatellite instability (MSI-H) because these regions of the genome are particularly unstable and susceptible to errors that do not get corrected because of the defective MMR system [2]. For determining MSI status, the National Cancer Institute (NCI) recommends a microsatellite panel (NCI panel) consisting of two mononucleotide repeats (BAT26 and A4725) and three dinucleotide repeats (D5S346, D2S123, and D17S250). Using the NCI panel, MSI-H tumors are defined as having instability in two or more markers, and tumors with low or stable microsatellite instability have instability in one or no markers [3]. Tumor MMR status is also determined by immunohistochemical (IHC) analysis of the protein products of genes involved in DNA MMR, such as *MLH1*, *MSH2*, and *MSH6*, and *PMS2* [4].

In advanced stage CRC patients, the fluoropyrimidine 5-fluorouracil (5FU) has been the most widely used chemotherapeutic agent since the late 1950s [5]. 5FU, alone or in combination with other drugs, is recommended for first-line treatment in Stage III, Stage IV, and high risk Stage II CRC [6]. Recently, there has been some suggestion that MSI-H status, from either hereditary or acquired causes, could potentially guide treatment decisions for this very commonly used chemotherapeutic agent [7]. The expected effect of using MSI status to guide treatment decisions is to withhold treatment with 5FU for individuals who are unlikely to respond or to use alternative chemotherapies. Clinical practice guidelines provide conflicting recommendations about the use of MSI status to guide 5FU treatment decisions. Three major cancer guideline groups have stated that use of MSI for predicting response to therapy is not recommended [8-10]. Alternatively, updated guidelines for 2013 from the National Comprehensive Cancer Network recommend MSI testing for all patients with Stage II disease because MSI positive patients may have a good prognosis and do not benefit from 5FU therapy [6].

**Figure 1:**
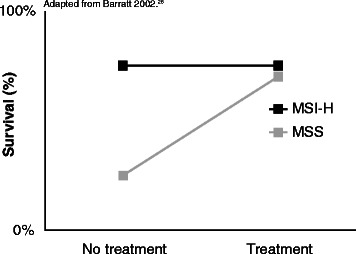
**MSI status is a prognostic and predictive marker.** Adapted from Barratt 2002 [26].

There is substantial data on the relationship between MSI status and prognosis in CRC patients. A meta-analysis with 7,642 cases demonstrated that patients with MSI-H tumors have a significantly better prognosis than those with MSS tumors [7]. This prognostic benefit of MSI-H status can confound the relationship between MSI and treatment effects, especially when only treated patients are considered. Figure 1 demonstrates that MSI-H patients appear to have better survival, even in the absence of a treatment benefit; their improved survival comes from the prognostic benefit of their MSI status rather than an effect of treatment.

We systematically reviewed the evidence for the interaction of microsatellite instability status (MSI) and treatment with 5FU in colorectal cancer to determine how well MSI status predicts health outcomes in patients undergoing 5FU-based chemotherapy.

## Methods

### Eligibility criteria

We considered studies that included both CRC patients treated with 5FU-based chemotherapy and untreated patients with survival outcomes presented by MSI status. We accepted either MSI or IHC testing to indicate MSI status. While any treatment containing 5FU therapy was included, we required that >90% of patients received 5FU in the treatment group for inclusion of the study. Eligible study designs included randomized controlled or controlled clinical trials and prospective or retrospective cohort studies. We excluded studies that did not report disease free survival (DFS) or overall survival (OS) outcome separately for patients based on MSI and treatment status (or where data could not be calculated).

### Identification of studies

Systematic literature searches were performed in the following databases through June 27, 2013: MEDLINE, PubMed, Cochrane Database of Systematic Reviews, and Cochrane Central Register of Controlled Trials. The search strategy was built to comprehensively capture the literature related to MSI and chemotherapeutic agents in the context of CRC treatment. Search details are provided in the supplementary materials (see Additional file 1: Search Strategy). The database searching was supplemented by the review of reference lists from existing reviews, hand searching selected scientific conferences from 2010-2013, and a search of ongoing and recently completed studies on clinicaltrials.gov.

Identified abstracts were independently reviewed by two investigators against inclusion criteria specified *a priori*. Full-text articles of abstracts meeting inclusion criteria were dual-reviewed against the inclusion criteria. Disagreements were resolved through consensus or the input of a third reviewer.

We reviewed studies for the independence of their study populations. In the case where multiple studies were identified with population overlap, we included the study that presented the needed data with the largest number of participants.

### Quality assessment and data abstraction

At least two investigators independently assessed the quality of each included study using questions adapted from the QUADAS-2 (Table 1) [11]. Overall quality codes were determined by discussion and consensus between the two reviewers. Quality categories of good, fair+, fair-, and poor were used to indicate the relative risk of bias or concerns about applicability of each study. Despite the limitations of categorizing studies in this manner [12], these categories were used for ease of grouping. Studies that were considered poor quality were excluded from the analysis.

**Table 1:** **Article quality rating**

Quality rating questions	Quality categories
• Were the test(s) clearly described (number of loci tested, MMR genes, etc?) AND did the Index test(s) meet NIH standards?	• Good: Studies with a low risk of bias and minimal concerns of applicability
• Was the spectrum of patients/tumors representative of the patients/tumors who will receive the test in practice?	• Fair+: Studies with some risk of bias or concerns regarding applicability; testing does meet NIH standards
• Was the patient (sample) selection process from the source population (retrospective studies) clearly described? If prospective, were patient selection criteria clearly described?	• Fair -: Studies with some risk of bias or concerns regarding applicability; testing does not meet NIH standards
• In a retrospective study, were selected samples representative (50% of original sample number; not statistically different on key characteristics e.g. stage distribution) of the original complete sample set?	• Poor: Studies with a significant risk of bias or greater concerns regarding applicability
• Were patient withdrawals (prospective) or sample losses (retrospective) from the source population explained?	
• Were un-interpretable, indeterminate, or intermediate test results reported? (Includes samples with insufficient DNA)	
• Was follow-up sufficiently long? (minimum 3 years)	
• If prospective, was treatment assignment blinded to MSI status?	

Data related to study design, population characteristics, chemotherapy use, MSI testing method and completeness, and relevant outcomes were abstracted from included studies into evidence tables with dual data checking by a second reviewer. Disagreements were resolved through consensus or input of a third reviewer.

Studies were included in the analysis regardless of stage at diagnosis for the patients. We classified the studies as ‘advanced stage’ or ‘mixed stages.’ Advanced stage refers to study populations in which all patients had metastatic CRC, and distant metastases. Mixed stages refers to studies with a combination of patients from Stage I to Stage IV at diagnosis.

Testing was classified by ‘completeness.’ MSI testing was considered complete if at least the 5 marker NCI panel was tested according to NCI standards. Testing using fewer markers was considered incomplete. For IHC, testing was considered complete if the presence of proteins for all four genes (*MLH1, MSH2, MSH6, and PMS2)* was evaluated, and incomplete if one to three proteins were tested.

Regimens that included 5FU alone or with leucovorin only were classified as single agent treatment. Regimens that included 5FU in combination with other therapies, such as (but not limited to) FOLFOX, FOLFIRI, or CAPOX were classified as multi-drug treatment.

Studies were classified depending on the type of cancer for the patient, including colon only, rectal only, and colon/rectal combined.

When possible, we recorded outcomes data from multivariate analyses that adjusted for other potentially confounding variables. If only univariate analysis was reported, we recorded these results instead, but also retained an indicator of which analysis is reported.

### Statistical analysis

The hazard ratio (HR) and 95% confidence interval was abstracted from each article where possible. In cases where the HR was not reported, the value was estimated using the methods described by Tierney et al. [13] HR analysis involved transforming data to ensure that for all studies a HR lower than 1 referred to improved survival in the group receiving 5FU chemotherapy compared to the group receiving no treatment. In general, data associated with the longest follow-up was retained; however, in instances where patient groups within the same study had variable lengths of follow up, estimates were made for all groups at the shortest time point to allow all groups equivalent time to accrue events.

We conducted random-effects meta-analyses to estimate the pooled effect size of disease-free survival (DFS) and overall survival (OS). We used the metan procedure in Stata 11.2® [14] for all meta-analyses, which implements the DerSimonion & Laird method [15]. Analyses were stratified by patient MSI status (MSI-H versus MSS). To examine the statistical significance of the pooled estimates in different strata, we calculated the difference in the log of the pooled effects and their standard error, and from those statistics calculated a z-score and related p-values. Because the DerSimonian & Laird methods can underestimate statistical heterogeneity when heterogeneity is high or few studies are being pooled, we ran sensitivity analyses using the Profile Likelihood estimation method [16]. Results were almost identical between the two methods, so DerSimonian & Laird results are presented in the text and figures.

We examined the I^2^ statistic as a measure of statistical heterogeneity. We applied the Cochrane Collaboration’s rules of thumb for interpreting I^2^: less than 40 percent likely represents unimportant heterogeneity, 30 to 60 percent represents moderate heterogeneity, 50 to 90 percent represents substantial heterogeneity; above 75 percent indicates considerable heterogeneity among the studies [17]. We did not conduct any analyses to assess the presence of publication bias or selective outcome reporting within our included studies.

DFS is defined in various ways throughout the CRC literature. Measures of DFS vary in their beginning time point (diagnosis, randomization, and enrollment) and included endpoints (recurrence, progression, and death). In addition to studies of DFS, other studies reported related surrogate endpoints including recurrence-free survival or time to recurrence. Similar variation exists in the measurement of overall survival (OS) in oncology literature. Some studies of survival include only cancer-specific deaths while others include all-cause deaths. It is unclear whether this lack of consistency between trials may alter the findings of a systematic review [18]. In order to examine the impact of these heterogeneous endpoints, multiple sensitivity analyses were conducted from least to most restrictive definition for DFS and OS. The most restrictive were based on definitions published by the FDA in 2007 for guiding clinical trial endpoints [19]. We also performed a subset analysis to assess the study findings in stage II only.

## Results

### Eligible studies

**Figure 2:**
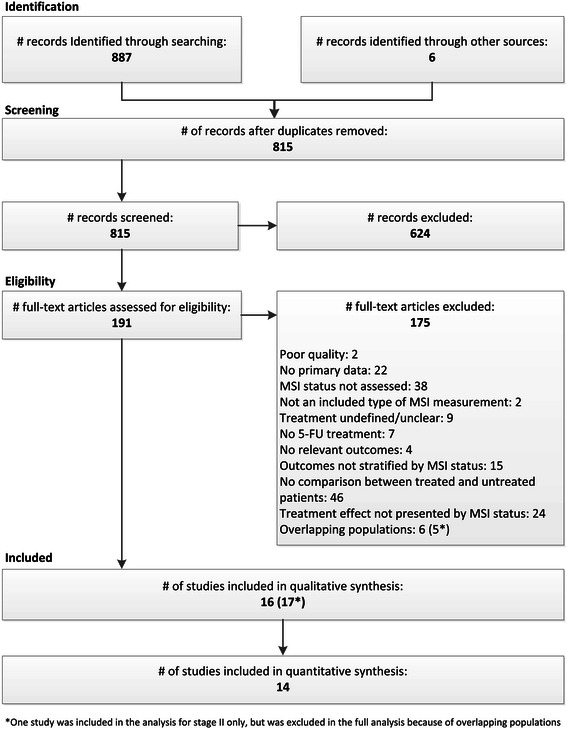
**Literature flow diagram.**

The literature search identified 815 articles for evaluation against the eligibility criteria. We excluded 624 articles based on review of the abstract, and retrieved and evaluated 191 full text articles. After review, 16 studies were included for qualitative analysis and 14 studies were eligible for meta-analysis (see Excluded Studies list in Additional file 1: Search Strategy). The Literature Flow Diagram is presented in Figure 2.

We excluded 46 studies that only reported on subjects treated with 5FU (with no comparison to an untreated population) that would have otherwise been included. For DFS, there were 6 articles that were retained for the meta-analysis of HR [20-25]. For OS there were 14 articles that were retained for the meta-analysis of HR [20-33]. Two studies [34,35] are included in qualitative discussion but they did not provide the necessary data to be included in the meta-analysis.

All papers were rated as fair quality with the exception of one paper [33] that was rated as good quality. Two papers were excluded from this review based on poor quality [36,37]. Lukish et al. [36] was considered poor quality because the MSI testing was not performed according to the NCI standards [3], all patients were less than 40 years old so the population was not representative of a general population, and the study had a small sample size. Bertagnolli et al. [37] was considered poor quality because the treated and untreated groups were decided by stage and therefore the difference in outcomes is likely highly confounded in terms of prognosis. The fair quality papers were divided into two groups based on whether the MSI testing met NCI standards [20,21,23,25,28-30,34,35] or not [22,24,26,27,31,32].

### Microsatellite instability (MSI)

Overall, nine studies were classified as having complete testing for MSI status [20,21,23,25,28,29,33-35]. Ten studies used MSI testing only [20,23,25-29,33-35], 4 studies used IHC testing only [24,30-32], and 2 studies used both MSI and IHC testing to determine MSI status [21,22]. For the purposes of this analysis, subjects were classified as either MSI high (MSI-H) or MSI low or stable (MSS). The mean percentage of MSI-H subjects was 15% for the included studies, which is comparable to the expected percentage of MSI-H based on population estimates [38].

### Study characteristics

Characteristics of the eligible studies are provided in Table 2. There were a total of 9,312 subjects included across all studies. All of the studies included for the final analysis used treatment regimens that included 5FU as a single agent. One study also included a separate analysis of a small subset of patients (n = 16) that received 5FU, leucovorin, and oxaliplatin (FOLFOX); however, this sample size is too small to draw a meaningful conclusion [32].

**Table 2:** **Study characteristics**

		Study sample characteristics				5FU Treatment regimen	MSI assessment			Included in HR meta-analysis	
Study	N	Tumor	Stage	% stage II	% stage IV		MSI test version	MSI test completeness	MSI-H (%)	DFS	OS
Hong 2012 [12]	947	Colorectal	Mixed	40%	16.9%	NR	MSI	Complete	9%	X^2^	X^2^
Hutchins 2011 [11]	1913	Colorectal	Mixed	89%	0%	Single	IHC	Partial	11%	X^1^	X^1^
Jover 2009 [21]	496	Colorectal	Mixed	59%^3^	0%	Single	MSI+IHC	Complete	12%	X^2^	X^2^
Kim 2007 [25]	542	Colon	Mixed	NR	0%	Single	MSI	Complete	18%	X^1^	X^1^
Sargent 2010 [22]	1027	Colon	Mixed	52%	0%	Single	MSI+IHC	Partial	16%	X^2^	X^2^
Storojeva 2005 [23]	160	Colorectal	Mixed	NR	NR	Single	MSI	Complete	13%	X^2^	X^2^
Barratt 2002 [26]	368	Colon	Mixed	61%^3^	NR	Single	MSI	Partial	24%		X
Benatti 2005 [28]	1263	Colorectal	Mixed	58%^3^	14.6%	Single	MSI	Complete	20%		X
Carethers 2004 [29]	204	Colorectal	Mixed	52%	0%	Single	MSI	Complete	18%		X^1^
Elsaleh 2001 [27]	732	Colorectal	Mixed	0%	0%	Single	MSI	Partial	9%		X
Lanza 2006 [30]	325	Colorectal	Mixed	0%	0%	Single	IHC	Partial	13%		X^1^
Liang 2002 [33]	244	Colorectal	Advanced	0%	100%	Single	MSI	Complete	21%		X^1^
Ohrling 2010 [31]	718	Colorectal	Mixed	50%	0%	Single	IHC	Partial	20%		X^1^
Wangefjord 2013 [32]	112	Colorectal	Mixed	0%	0%	Single	IHC	Partial	15%		X^1^
Colombino 2002 [35]	91	Rectal	Mixed	42%	NR	Single	MSI	Complete	19%		
Dietmaier 2006 [34]	170	Colon	Mixed	0%	0%	Single	MSI	Complete	14%		
Ribic	570	Colon	Mixed	55%	0%	Single	MSI	Partial	17%		
**Total**	**9312**								**15%**		

(5FU = 5 fluorouracil; DFS = disease free survival; OS = overall survival; HR = hazard ratio; NR = not reported; MSI = microsatellite instability; IHC = immunohistochemistry; X = study was included in the analysis).

^1^Hazard ratio had to be estimated from study (see Methods).

^2^Multivariate hazard ratio used in analysis.

^3^Percentage stage II estimated from data reported on full population prior to exclusions for the final analysis data set.

Studies were included in the analysis regardless of patient stage at diagnosis. Although in most cases the proportion of patients at each stage was reported, results were not stratified by stage, so results are reported for all stages combined. An analysis focused on stage II cases is presented after the results that included all stages combined. Most studies included both rectal and colon cancer.

### MSI status and effect of 5FU treatment based on disease free survival (DFS)

**Figure 3:**
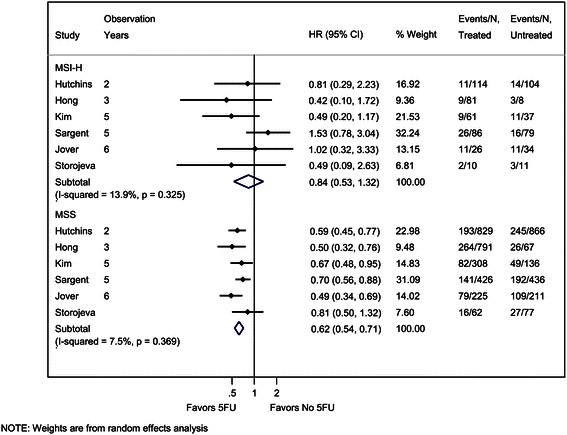
**Forest plot of hazard ratios (HRs) for the effect of 5FU treatment on disease-free survival (DFS) by MSI status.** By convention, ratios less than 1.0 indicate longer DFS for patients who receive 5FU treatment compared with untreated patients. The test of significance for the difference in the HR for the MSI-H versus MSS groups was not statistically significant (p = 0.111).

Forest plots of the effect of 5FU treatment on DFS by MSI status are shown using HRs (Figure 3). For the MSS group, there was an effect of 5FU treatment on DFS with summary HR of 0.62 (95% CI: 0.54, 0.71), indicating that *patients who received 5FU treatment had longer DFS than patients who did not receive this treatment*. We did not detect evidence of heterogeneity across studies for the MSS group for the HR analysis (I^2^ = 7.5%, p = 0.37).

In contrast, the effect of 5FU treatment on DFS was not statistically significant for MSI-H patients with a pooled HR of 0.84 (95% CI: 0.53, 1.32). We did not detect evidence of heterogeneity across studies for the MSI-H group (I^2^ = 13.9%, p = 0.33). The sample size for the MSI-H group is smaller than the sample size for the MSS group (651 vs. 4434), and the confidence interval for each study is wider for the MSI-H group compared with the MSS group.

Comparing the summarized point estimates of the effect of 5FU treatment for the MSS group to the MSI-H group, there is not a statistically significant difference (p = 0.11). *Thus, we did not find evidence that MSI status affects the likelihood of DFS with 5FU treatment, however data were limited.*

One study [35] reported on DFS but could not be included in the quantitative synthesis because it was not possible to estimate the HR using the data available in the publication. In this study, the median DFS was very similar for treated and untreated patients with MSI-H status (34 months versus 36 months, respectively), and for patients with MSS status (24 months versus 25 months, respectively).

We conducted sensitivity analyses to determine whether the results of the analysis are affected by the definition of the DFS outcome. The primary analysis we presented above is the broadest and most inclusive, and incorporated all studies that presented a HR of anything described by the authors as DFS or regression free survival (RFS), which could include substantially different definitions. In sensitivity analyses, we restricted the analysis only to studies that include death as an event for DFS and used the FDA definition for DFS [21,22]. When only these two studies are included, there is a statistically significant difference in the effect of 5FU treatment on DFS based on MSI status (p = 0.008). The pooled HRs were quite different, however data was limited to only two studies with only 225 persons with MSI-H (MSI-H:pooled HR = 1.38 (95% CI: 0.77 – 2.49), MSS: HR = 0.60 (95% CI: 0.42 – 0.85), data not shown).

### MSI status and effect of 5FU treatment based on overall survival (OS)

**Figure 4:**
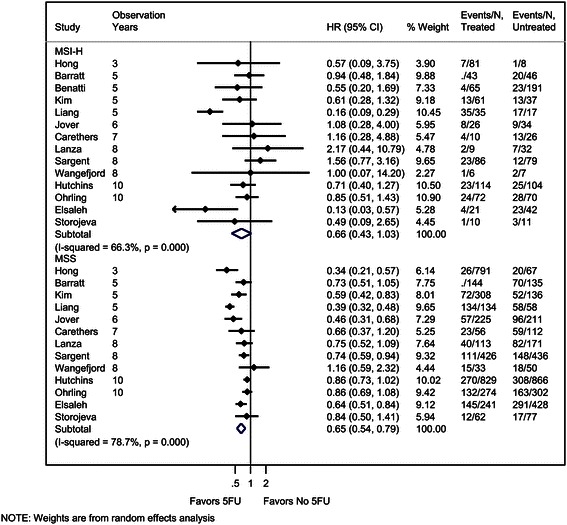
**Forest plot of hazard ratios (HRs) for the effect of 5FU treatment on overall survival (OS) by MSI status.** By convention, ratios less than 1.0 indicate longer OS for patients who receive 5FU treatment compared with untreated patients. The test of significance for the difference in HR for MSI-H versus MSS groups was not statistically significant (p = 0.45). Benatti et al. [28] was included in the figure and point estimate, but this study was removed for calculation of p-values, since it contributed data only to the MSI-H group.

Forest plots of the effectiveness of 5FU treatment on OS by MSI status are shown using the HR (Figure 4). 5FU treatment was effective in increasing OS in the MSS group with a summary HR of 0.65 (95% CI: 0.54, 0.79), indicating that patients who received 5FU treatment had longer OS than patients who did not receive this treatment. There was evidence of substantial heterogeneity across studies for the MSS group (I^2^ = 79%, p < 0.001). The largest estimate of the effect of treatment was seen in the study with the shortest followup [20]. In general, there appeared to be a trend of decreasing benefit as the years of observation increased.

The effect of 5FU treatment on OS was very similar for MSI-H patients with a pooled HR of 0.66 (95% CI: 0.43, 1.03), although the difference between those treated and not treated with 5FU was not statistically significant in this group. There was evidence of substantial heterogeneity across studies for the MSI-H group (I^2^ = 66%, p < 0.001). This heterogeneity across studies may be driven by lower estimates of the effect of treatment for two studies [27,33], and in general there were relatively small sample sizes with fewer than 100 observations per study in most cases. The confidence intervals around the point estimates for each study are wider for the MSI-H group compared with the MSS group, reflecting the smaller number of patients with MSI-H status (e.g., overall N = 1293 vs. 6685 for MSI-H and MSS, respectively).

Not surprisingly, the pooled estimates the effect of 5FU treatment were not statistically different between the two groups (p = 0.45). *Thus, it is unlikely the 5FU treatment has a differential effect on OS based on MSI status, however uncertainty remains due to the relatively few events in the MSI-H group and the wide variability in HRs in that group.* We note that effect sizes were smaller for MSI-H than MSS patients in all but two of the studies with follow-up of 8 years or less, though differences were not statistically significant. Given the small number of events, these results are consistent with either a reduced likelihood of improved OS for MSI-H patients or no difference between groups.

Two studies [34,35] reported on OS that could not be included in the quantitative synthesis because it was not possible to estimate the HR using the data available in the publications. In one study, the median OS was very similar for treated and untreated patients with MSI-H status (both 37 months), and for patients with MSS status (26 months versus 28 months, respectively). In the second study, patients who received treatment with 5FU had fewer events compared with untreated patients for MSI-H (OR = 0.40; 95% CI: 0.07 – 2.34) and MSS (OR = 0.45; 95% CI: 0.23 – 0.90) patients, with a median follow-up of 44.5 months.

As with DFS, we conducted sensitivity analyses to determine whether the results of the analysis were affected by definition of the OS outcome. In sensitivity analyses, we used increasingly restrictive definitions: 1) only studies that use all-cause mortality [20-23,25,26,31,33] 2) only studies that use cancer-specific survival [27,28,32] and 3) only studies that use the FDA definition of OS [21,22,24,26,33]. These analyses did not change the findings, except that in the analysis of studies that use cancer-specific survival the pooled HR estimate for the MSS group was no longer statistically significantly different from 1.0, presumably because of the small sample size with only two studies contributing to the analysis [data not shown].

### MSI status and effect of 5FU treatment in stage II cases only

We assessed the relationship between MSI status and treatment with 5FU in stage II cases only, because the NCCN guidelines recommend MSI testing for treatment decisions only in this group [6]. There was very limited data available to address this question, since most published studies conducted a joint analysis of mixed stages, and did not provide stratified data by stage. We found three studies that provide data on stage II cases only with a total of 1319 subjects [22,28,39], and one study where the study population included a very high percentage (91%) of stage II cases with a total of 1708 subjects (Table 2) [24]. Because the data did not use consistent measures across studies, we did not perform a meta-analysis. Instead, we provide a qualitative description of the results.

Ribic et al. [39] reported no difference in OS in MSS patients (HR = 0.67; 95% CI 0.39 – 1.15), or in MSI-H patients (HR = 3.28; 95% CI 0.86 – 12.48) with 7.4 years of follow-up. Sargent et al. [22] reported similar findings for DFS, with no difference in DFS for MSS patients (HR = 0.84; 95% CI 0.57 – 1.24; p = 0.38) or for MSI-H patients (HR = 2.30; 95% CI 0.84 – 6.24; p = 0.09) depending on treatment status. For the OS outcome, they reported that the conclusions were similar (data not shown) except that for MSI-H patients the 5FU treated group had decreased OS compared with surgery alone (HR = 2.95; 95% CI 1.02 – 8.54; p = 0.04). Benatti et al. [28] reported no difference in OS for MSS patients (log rank = 2; p = 0.151) or for MSI-H patients (log rank = 0.97; p = 0.323) depending on 5FU treatment status with 5 years of follow-up. Hutchins et al. [24] reported a difference in DFS for MSS patients (HR 0.59; 95% CI 0.45 – 0.77), but not for MSI-H patients (HR = 0.81; 95% CI 0.29 – 2.33) with 10 years of follow-up [Figure 2]. For OS, there was not difference in either group based on MSI status [Figure 3]. Consistent with the findings for all stages combined, these studies suggest a potential trend towards larger beneficial effects of treatment in MSS patients compared with MSI-H patients, although the differences are not statistically significant.

In order to incorporate data from as many studies as possible, we also considered all of the studies ranked by percentage of stage II patients. We did not observe a pattern suggesting a relationship between the percentage of stage II patients and response to 5FU by MSI status (see Additional file 2: Figure S1 & Additional file 3: Figures S2).

## Discussion

We assessed MSI status as a predictive marker for health outcomes in patients undergoing treatment with 5FU-based chemotherapy. The results of this meta-analysis showed that treatment with 5FU-based chemotherapy improved DFS and overall survival in MSS patients and there was a statistically non-significant trend towards better survival in MSI-H patients with 5FU treatment, and no clear difference in the effect of treatment based on MSI status.

Our results expand upon the results of a previous meta-analysis of the same topic by Guastadisegni et al. [40], which included a total sample size of 3121, compared with 9312 subjects in the present analysis. All of the studies reported in the previous analysis are included in our analysis, with the exception of one study [39] that had an overlapping population with a larger and more recent study that was included in our analysis [22]. The previous analysis also differed from ours in the statistical procedures used to compute the summary statistic. We used methods to estimate the HR [13], which takes into account the number and timing of events, and censoring. Odds ratios (ORs) take into account only the number of events, and can produce biased estimates for time-to-event outcomes. The authors of the previous analysis on this topic concluded that whereas they could establish MSI status as a favorable *prognostic* marker based on DFS (summary OR = 0.58, 95% CI 0.47 – 0.72) and OS (summary OR = 0.6; 95% CI 0.53 – 0.69) for CRC patients, they had inconclusive results for MSI status as a *predictive* marker of response to treatment with 5FU chemotherapy. Similar to our analysis, they determined that there was a significant beneficial effect of 5FU chemotherapy in patients with MSS tumors (OR = 0.52; 95% CI 0.4 – 0.6). However, the sample size for patients with MSI-H tumors in their analysis was small (n = 396). The point estimate for the effect of 5FU treatment in MSI-H patients was similar to what we found (OR = 0.69; 95% CI 0.3 – 1.5) for OS, and there was significant heterogeneity across studies (p = 0.03), so no clear conclusion could be reached. Our analysis included a larger population of 1293 MSI-H patients; however, statistically significant heterogeneity was still identified in the overall survival analysis.

Des Guetz et al. [41] also reported a meta-analysis on this topic that included seven studies with 3690 patients combined, six of which were reported by Guastadisegni et al. [40] All patients included in these studies are Stage II or Stage III CRC cases. Given this overlap, not surprisingly they found a beneficial effect of chemotherapy among MSS patients, but no benefit of 5FU chemotherapy among MSI-H patients for Regression-Free Survival (HR = 0.96; 95% CI 0.62 – 1.49) or OS (HR = 0.70, 95% CI 0.44 – 1.09). In contrast to the conclusions of Guastadisegni et al. [40], these authors concluded that MSI-H status is a predictive factor of non-response to 5FU chemotherapy based on a meta-analysis for the interaction between MSI status and treatment effect, suggesting a lesser benefit for MSI-H patients compared with MSS patients ((HR = 0.77, 95% CI 0.68 – 0.87).

A previous study addressed this question and reached seemingly different conclusions. Des Guetz et al. [42] conducted a meta-analysis restricted only to patients with metastatic CRC who received treatment with 5FU chemotherapy. The results were reported in terms of the response rate ratio using the RECIST criteria [43]. The authors found that among patients treated with 5FU chemotherapy, the response rate ratio was 0.82 (95% CI 0.65 – 1.03), where ratios less than 1.0 indicate that the response rate to chemotherapy was better among MSI-H patients compared with MSS patients. However, the authors concluded that MSI status does not predict the effect of 5FU chemotherapy. When we repeated this analysis with existing studies [33,44-54], however, we had a statistically significant finding. For studies in which patients were treated with 5FU as a single agent, the summary RR is 0.52 (95% CI 0.40 – 0.68), indicating that MSI-H patients have a *better* response to 5FU chemotherapy than MSS patients (see Additional file 4: Figure S3). This apparent discrepancy can be understood by considering Figure 1, which shows the expected impact of MSI status as *both* a prognostic and predictive factor. By evaluating only patients who received 5FU treatment, we cannot untangle the effects of prognosis and treatment. Interestingly, in this analysis we observed a substantially different effect for studies in which patients were treated with 5FU as a single agent versus studies in which patients received 5FU as part of a combination therapy. Unfortunately, none of the studies in our primary meta-analysis used combination therapy, so we were unable to confirm whether this effect remained using different outcomes (DFS, OS) or subjects with different stages of CRC.

The data that we report do not support the recommendation for determination of MSI status in stage II CRC patients. One guideline recommends determination of MSI status for all patients with stage II disease because stage II MSI-H patients may have good prognosis and do not benefit from 5FU treatment [6]. This guideline only cites the results of one study [22], which was also reported by us, showing a statistically significant decrease in OS in the treatment group compared with surgery alone in MSI-H stage II patients (HR = 2.95; 95% CI 1.02 – 8.54; p = 0.04). This was the only study with a statistically significant finding; the others showed no difference for stage II patients in MSI-H patients. Further, this study had the most extreme HR in favor of no 5FU treatment for MSI-H patients among studies that reported on stage II patients (Additional file 2: Figure S1 and Additional file 3: Figure S2).

There were several limitations to our meta-analysis. First, not all studies reported hazard ratios or counts of events, which were the primary data necessary to include the studies in the meta-analysis. In some cases, we were able estimate the HR value using the methods described by Tierney et al. [13]. We conducted a sensitivity analysis by excluding studies in which the data were estimated, and the overall conclusions remained the same (data not shown). Related to this issue, a second limitation of our approach is that when we estimated data using the Kaplan-Meier curves, we selected the longest possible follow-up available to determine the estimated number of events. This approach could lead to potential bias towards the null hypothesis of no difference, because Figures 3 and 4 show a possible trend with the most significant HRs being the ones with the shortest follow-up time.

None of the studies reported in our primary meta-analysis used contemporary treatment regimens that include 5FU in combination therapies, such as FOLFOX or FOLFIRI. This is a strength of the available data, since it is then possible to explore the ability of MSI status to predict 5FU treatment response in isolation, and without contamination of the effects of other treatments given simultaneously. However, it is not clear that these findings represent the effect of MSI status on response to 5FU in combination therapy.

We did not consider whether molecular subtypes of MSI-H patients experience differential response to 5FU chemotherapy. Greater heterogeneity of MSI-H tumors is now recognized [55] with various epigenetic and genetic alterations that may alter the prognostic and predictive implications for patients. For instance, recently, large deletions in HSP110 T_17_ have been associated with improved survival in patients treated with 5FU-based chemotherapy [56,57]. Future analyses may need to account for this heterogeneity.

Finally, despite the increase in sample size for this analysis compared with previously reported meta-analyses on this topic, the number of MSI-H patients available for analysis remains limited, with substantially larger confidence intervals and lower statistical power compared with the findings for the MSS group. This limitation is coupled with an evidence base that relies primarily on retrospective study designs that have a greater potential for selection bias, and substantial (although not significant) heterogeneity of effect sizes reported in studies. One ongoing prospective study was identified in our search for additional clinical trials and meeting abstracts: The Eastern Cooperative Oncology Group (ECOG) E5202 Trial (ClinicalTrials.gov Identifier: NCT00217737) will assign treatment arms for stage II colorectal cancer patients based on MSI status in conjunction with 18q LOH. Those patients who are considered high risk (based on MSS and 18q LOH) will be randomized to FOLFOX6 with or without bevacizumab. Patients considered low risk due to retention of 18q alleles or MSI-H status will receive observation alone. While no publications were identified for this study, the clinical trial record indicates that primary data collection is completed.

## Conclusions

We conclude that the results of this meta-analysis do not support the use of MSI status for the direct benefit of the patient in guiding decisions about the use of 5FU treatment for CRC. Testing for MSI status among CRC patients is becoming increasingly common [58], given efforts to implement recommendations for universal screening of newly diagnosed CRC patients to identify cases of Lynch Syndrome [59]. Whereas substantial benefit of MSI testing has been demonstrated in terms of impact on relatives [60] and impact on prognosis [7], there remains limited use of this testing in guiding treatment decisions for patients already diagnosed with CRC.

## Additional files

Additional file 1:
**Search strategy used to identify relevant studies in Medline.**

Additional file 2: Figure S1. Forest plot of hazard ratios (HRs) for the effect of 5FU treatment on disease-free survival (DFS) by MSI status ranked by percentage of stage II patients. By convention, ratios less than 1.0 indicate longer DFS for patients who receive 5FU treatment compared with untreated patients. The test of significance for the difference in the HR for the MSI-H versus MSS groups was not statistically significant (p = 0.111).
Additional file 3: Figure S2. Forest plot of hazard ratios (HRs) for the effect of 5FU treatment on overall survival (OS) by MSI status ranked by percentage of stage II patients. By convention, ratios less than 1.0 indicate longer OS for patients who receive 5FU treatment compared with untreated patients. The test of significance for the difference in HR for MSI-H versus MSS groups was not statistically significant (p = 0.45). Benatti et al. [28] was included in the figure and point estimate, but this study was removed for calculation of p-values, since it contributed data only to the MSI-H group.
Additional file 4: Figure S3. Forest plot of response rate ratio (RR) for the effect of MSI status on response to treatment among patients treated with 5FU therapy. By convention, ratios less than 1.0 indicate a better response to treatment among patients with MSI-L/S status.
