# Supplementary material for: Systematic review of the predictive effect of MSI status in colorectal cancer patients undergoing 5FU-based chemotherapy
Source: BMC Cancer. 2015 Mar 21;15:156. doi: 10.1186/s12885-015-1093-4 (PMC4376504; doi:10.1186/s12885-015-1093-4)
Supplement: Additional file 1: — Search strategy used to identify relevant studies in Medline. [file 12885_2015_1093_MOESM1_ESM.doc]

**Additional file 1: Search Strategy**

Database: Ovid MEDLINE(R) In-Process & Other Non-Indexed Citations <June 26, 2013>

Search Strategy:

--------------------------------------------------------------------------------

1 Colorectal Neoplasms/ (0)

2 Colonic Neoplasms/ (0)

3 Sigmoid Neoplasms/ (0)

4 Rectal Neoplasms/ (0)

5 Anus Neoplasms/ (0)

6 Anal Gland Neoplasms/ (0)

7 CRC.ti,ab. (1088)

8 mCRC.ti,ab. (101)

9 ((colon or colorectal or colonic or sigmoid or rectal or rectum or anus or anal) adj3 (cancer* or neoplasm* or neoplasia* or tumor* or tumour* or carcinoma* or adenocarcinoma* or metastatic or metastasis or metastases)).ti,ab. (7493)

10 or/1-9 (7598)

11 Genomic instability/ (1)

12 Microsatellite instability/ (0)

13 DNA repair/ (0)

14 DNA mismatch repair/ (0)

15 Microsatellite Repeats/ (0)

16 (genom* adj stabil*).ti,ab. (268)

17 (genom* adj instabil*).ti,ab. (402)

18 microsatellite.ti,ab. (1940)

19 dna repair*.ti,ab. (1238)

20 (excision adj2 repair*).ti,ab. (350)

21 mismatch repair*.ti,ab. (272)

22 replication error*.ti,ab. (29)

23 MSI.ti,ab. (242)

24 MSS.ti,ab. (113)

25 11 or 12 or 13 or 14 or 15 or 16 or 17 or 18 or 19 or 20 or 21 or 22 or 23 or 24 (4221)

26 Antineoplastic Agents/ (1)

27 antineoplastic*.ti,ab. (477)

28 (antitumo?r adj drug*).ti,ab. (158)

29 (anti-tumo?r adj drug*).ti,ab. (34)

30 (antitumo?r adj agent*).ti,ab. (251)

31 (anti-tumo?r adj agent*).ti,ab. (63)

32 Chemotherapy, Adjuvant/ (0)

33 Neoadjuvant Therapy/ (0)

34 Antineoplastic Combined Chemotherapy Protocols/ (1)

35 chemotherap*.ti,ab. (13818)

36 chemosensitiv*.ti,ab. (345)

37 Fluorouracil/ (0)

38 fluorouracil.nm. (0)

39 fluorouracil.ti,ab. (1071)

40 capecitabine.ti,ab. (277)

41 5FU.ti,ab. (54)

42 5-FU.ti,ab. (535)

43 FOLFOX.ti,ab. (110)

44 FOLFIRI.ti,ab. (65)

45 XELOX.ti,ab. (30)

46 CAPOX.ti,ab. (3)

47 Adrucil.ti,ab. (0)

48 Carac.ti,ab. (1)

49 Efudex.ti,ab. (0)

50 Fluoroplex.ti,ab. (0)

51 or/26-50 (15351)

52 10 and 25 and 51 (38)

53 limit 52 to english language (38)

54 remove duplicates from 53 (37)

**Supplemental Material: Excluded Studies**

| **Exclusion Codes** | |
| --- | --- |
| **E1** | Study relevance – no MSI status |
| **E2** | Setting – No treatment with 5FU-based chemotherapy |
| **E3** | Population – Not CRC |
| **E4** | Population – Laboratory or cell line only |
| **E5** | Study design |
| **E6** | No relevant outcomes |
| **E7** | Outcomes not stratified by MSI status |
| **E8** | Treatment unclear/undefined |
| **E9** | Not all patients in analysis received 5FU |
| **E10** | No comparison between treated and untreated patients (all patients treated) |
| **E11** | Added benefit of treatment not presented by MSI H/L |
| **E12** | Not an included form of MSI measurement |
| **E13** | Overlapping population |
| **E14** | PFS but not RECIST |
| **E15** | Poor quality KQ4 |

1. Aparicio T, Schischmanoff O, Poupardin C, et al. Deficient mismatch repair phenotype is a prognostic factor for colorectal cancer in elderly patients. Digestive & Liver Disease 2013 Mar;45(3):245-50. **E8**
2. Baba H, Watanabe M, Okabe H, et al. Upregulation of ERCC1 and DPD expressions after oxaliplatin-based first-line chemotherapy for metastatic colorectal cancer. Br J Cancer 2012 Dec 4;107(12):1950-5. **E1**
3. Barault L, Charon-Barra C, Jooste V, et al. Hypermethylator phenotype in sporadic colon cancer: study on a population-based series of 582 cases. Cancer Res 2008 Oct 15;68(20):8541-6. **E2**
4. Basso M, Strippoli A, Orlandi A, et al. KRAS mutational status affects oxaliplatin-based chemotherapy independently from basal mRNA ERCC-1 expression in metastatic colorectal cancer patients. Br J Cancer 2013 Jan 15;108(1):115-20. **E1**
5. Bavi P, Prabhakaran SE, Abubaker J, et al. Prognostic significance of TRAIL death receptors in Middle Eastern colorectal carcinomas and their correlation to oncogenic KRAS alterations. Molecular Cancer 2010;9:203. **E11**
6. Bendardaf R, Lamlum H, Ristamaki R, et al. Mismatch repair status is a predictive factor of tumour response to 5-fluorouracil and irinotecan chemotherapy in patients with advanced colorectal cancer. Tumour Biol 2007;28(4):212-20. **E11**
7. Bendardaf R, Lamlum H, Ristamaki R, et al. Oncoprotein Bcl-2 and microsatellite instability are associated with disease-free survival and treatment response in colorectal cancer. Oncol Rep 2008 Nov;20(5):999-1004. **E7**
8. Bendardaf R, Lamlum H, Ristamaki R, et al. Thymidylate synthase and microsatellite instability in colorectal cancer: implications for disease free survival, treatment response and survival with metastases. Acta Oncol 2008;47(6):1046-53. **E7**
9. Bertagnolli MM, Warren RS, Niedzwiecki D, et al. p27Kip1 in stage III colon cancer: implications for outcome following adjuvant chemotherapy in cancer and leukemia group B protocol 89803. Clin Cancer Res 2009 Mar 15;15(6):2116-22. **E10**
10. Bertagnolli MM, Niedzwiecki D, Compton CC, et al. Microsatellite instability predicts improved response to adjuvant therapy with irinotecan, fluorouracil, and leucovorin in stage III colon cancer: Cancer and Leukemia Group B Protocol 89803. J Clin Oncol 2009 Apr 10;27(11):1814-21. **E10**
11. Bertagnolli MM, Redston M, Compton CC, et al. Microsatellite instability and loss of heterozygosity at chromosomal location 18q: prospective evaluation of biomarkers for stages II and III colon cancer--a study of CALGB 9581 and 89803. J Clin Oncol 2011 Aug 10;29(23):3153-62. **E15**
12. Braun MS, Richman SD, Quirke P, et al. Predictive biomarkers of chemotherapy efficacy in colorectal cancer: results from the UK MRC FOCUS trial.[Erratum appears in J Clin Oncol. 2008 Sep 10;26(26):4363]. J Clin Oncol 2008 Jun 1;26(16):2690-8. **E10**
13. Braun MS, Richman SD, Thompson L, et al. Association of molecular markers with toxicity outcomes in a randomized trial of chemotherapy for advanced colorectal cancer: the FOCUS trial. Journal of clinical oncology : official journal of the American Society of Clinical Oncology 2009;27:5519-28. **E10**
14. Brueckl WM, Moesch C, Brabletz T, et al. Relationship between microsatellite instability, response and survival in palliative patients with colorectal cancer undergoing first-line chemotherapy. Anticancer Res 2003 Mar;23(2C):1773-7. **E10**
15. Cecchin E, D'Andrea M, Lonardi S, et al. A prospective validation pharmacogenomic study in the adjuvant setting of colorectal cancer patients treated with the 5-fluorouracil/leucovorin/oxaliplatin (FOLFOX4) regimen. Pharmacogenomics J 2012 Aug 7. **E10**
16. Chan JY, Ong CW, Salto-Tellez M. Overexpression of neurone glial-related cell adhesion molecule is an independent predictor of poor prognosis in advanced colorectal cancer. Cancer Science 2011 Oct;102(10):1855-61.  **E7**
17. Charara M, Edmonston TB, Burkholder S, et al. Microsatellite status and cell cycle associated markers in rectal cancer patients undergoing a combined regimen of 5-FU and CPT-11 chemotherapy and radiotherapy. Anticancer Res 2004 Sep;24(5B):3161-7. **E10**
18. Chua W, Goldstein D, Lee CK, et al. Molecular markers of response and toxicity to FOLFOX chemotherapy in metastatic colorectal cancer. Br J Cancer 2009 Sep 15;101(6):998-1004. **E10**
19. Cortejoso L, Garcia MI, Garcia-Alfonso P, et al. Differential toxicity biomarkers for irinotecan- and oxaliplatin-containing chemotherapy in colorectal cancer. Cancer Chemotherapy & Pharmacology 2013 Jun;71(6):1463-72. **E1**
20. Dahlin AM, Palmqvist R, Henriksson ML, et al. The role of the CpG island methylator phenotype in colorectal cancer prognosis depends on microsatellite instability screening status. Clin Cancer Res 2010 Mar 15;16(6):1845-55. **E11**
21. de Vos tot Nederveen Cappel WH, Meulenbeld HJ, Kleibeuker JH, et al. Survival after adjuvant 5-FU treatment for stage III colon cancer in hereditary nonpolyposis colorectal cancer. Int J Cancer 2004 Apr 10;109(3):468-71. **E7**
22. de Wit M, Belt EJ, Delis-van Diemen PM, et al. Lumican and Versican Are Associated with Good Outcome in Stage II and III Colon Cancer. Ann Surg Oncol 2012 Jun 19. **E7**
23. Demes M, Scheil-Bertram S, Bartsch H, et al. Signature of microsatellite instability, KRAS and BRAF gene mutations in German patients with locally advanced rectal adenocarcinoma before and after neoadjuvant 5-FU radiochemotherapy. Journal of Gastrointestinal Oncology 2013 Jun;4(2):182-92. **E6**
24. Des Guetz G, Mariani P, Cucherousset J, et al. Microsatellite instability and sensitivitiy to FOLFOX treatment in metastatic colorectal cancer.[Erratum appears in Anticancer Res. 2007 Sep-Oct;27(5b):3667]. Anticancer Res 2007 Jul;27(4C):2715-9. **E10**
25. Des Guetz G, Lecaille C, Mariani P, et al. Prognostic impact of microsatellite instability in colorectal cancer patients treated with adjuvant FOLFOX. Anticancer Res 2010 Oct;30(10):4297-301. **E10**
26. Donada M, Bonin S, Nardon E, et al. Thymidilate synthase expression predicts longer survival in patients with stage II colon cancer treated with 5-flurouracil independently of microsatellite instability. Journal of Cancer Research & Clinical Oncology 2011 Feb;137(2):201-10. **E10**
27. Donada M, Bonin S, Barbazza R, et al. Management of stage II colon cancer - the use of molecular biomarkers for adjuvant therapy decision. BMC Gastroenterology 2013;13:36. **E11**
28. Elsaleh H, Powell B, Soontrapornchai P, et al. p53 gene mutation, microsatellite instability and adjuvant chemotherapy: impact on survival of 388 patients with Dukes' C colon carcinoma. Oncology 2000;58(1):52-9. **E13**
29. Elsaleh H, Joseph D, Grieu F, et al. Association of tumour site and sex with survival benefit from adjuvant chemotherapy in colorectal cancer. Lancet 2000 May 20;355(9217):1745-50. **E13**
30. Elsaleh H. The microsatellite instability phenotype in human colorectal carcinoma: relationship to sex, age, and tumor site. Gastroenterology 2001 Jul;121(1):230-1. **E6**
31. Elsaleh H, Iacopetta B. Microsatellite instability is a predictive marker for survival benefit from adjuvant chemotherapy in a population-based series of stage III colorectal carcinoma. Clinical Colorectal Cancer 2001 Aug;1(2):104-9. **E13**
32. Elsaleh H, Cserni G, Iacopetta B. Extent of nodal involvement in Stage III colorectal carcinoma: relationship to clinicopathologic variables and genetic alterations. Diseases of the Colon & Rectum 2002 Sep;45(9):1218-22. **E7**
33. Farina-Sarasqueta A, van LG, Moerland E, et al. The BRAF V600E mutation is an independent prognostic factor for survival in stage II and stage III colon cancer patients. Ann Oncol 2010 Dec;21(12):2396-402. **E11**
34. French AJ, Sargent DJ, Burgart LJ, et al. Prognostic significance of defective mismatch repair and BRAF V600E in patients with colon cancer. Clin Cancer Res 2008 Jun 1;14(11):3408-15. **E10**
35. Garrity MM, Burgart LJ, Mahoney MR, et al. Prognostic value of proliferation, apoptosis, defective DNA mismatch repair, and p53 overexpression in patients with resected Dukes' B2 or C colon cancer: a North Central Cancer Treatment Group Study. J Clin Oncol 2004 May 1;22(9):1572-82. **E11**
36. Gavin P, Colangelo LH, Fumagalli D, et al. Mutation Profiling and Microsatellite Instability in Stage II and III Colon Cancer: An Assessment of their Prognostic and Oxaliplatin Predictive Value. Clin Cancer Res 2012 Oct 8. **E10**
37. Halling KC, French AJ, McDonnell SK, et al. Microsatellite instability and 8p allelic imbalance in stage B2 and C colorectal cancers. J Natl Cancer Inst 1999 Aug 4;91(15):1295-303. **E11**
38. Han SW, Lee HJ, Bae JM, et al. Methylation and microsatellite status and recurrence following adjuvant FOLFOX in colorectal cancer. Int J Cancer 2012 Oct 4. **E10**
39. Hemminki A, Mecklin JP, Jarvinen H, et al. Microsatellite instability is a favorable prognostic indicator in patients with colorectal cancer receiving chemotherapy. Gastroenterology 2000 Oct;119(4):921-8. **E10**
40. Hornberger J, Lyman GH, Chien R, et al. A multigene prognostic assay for selection of adjuvant chemotherapy in patients with T3, stage II colon cancer: impact on quality-adjusted life expectancy and costs. Value in Health 2012 Dec;15(8):1014-21. **E6**
41. Huang MY, Wang JY, Huang ML, et al. Polymorphisms in XPD and ERCC1 Associated with Colorectal Cancer Outcome. International Journal of Molecular Sciences 2013;14(2):4121-34. **E1**
42. Ide T, Kitajima Y, Ohtaka K, et al. Expression of the hMLH1 gene is a possible predictor for the clinical response to 5-fluorouracil after a surgical resection in colorectal cancer. Oncol Rep 2008 Jun;19(6):1571-6. **E12**
43. Ismail AS, Quinn MG, Wright MA, et al. A phase II and pharmacologic study of fluorouracil given by a 1-hour infusion daily for 5 days with leucovorin and interferon alpha-2a in adenocarcinoma of the large bowel. Oncol Rep 2005 Jun;13(6):1145-52. **E10**
44. Jensen LH, Danenberg KD, Danenberg PV, et al. Predictive value of MSH2 gene expression in colorectal cancer treated with capecitabine. Clinical Colorectal Cancer 2007 Mar;6(6):433-5. **E7**
45. Jensen SA, Vainer B, Kruhoffer M, et al. Microsatellite instability in colorectal cancer and association with thymidylate synthase and dihydropyrimidine dehydrogenase expression. BMC Cancer 2009;9:25. **E10**
46. Jover R, Zapater P, Castells A, et al. Mismatch repair status in the prediction of benefit from adjuvant fluorouracil chemotherapy in colorectal cancer. Gut 2006 Jun;55(6):848-55. **E11**
47. Jover R, Nguyen TP, Perez-Carbonell L, et al. 5-Fluorouracil adjuvant chemotherapy does not increase survival in patients with CpG island methylator phenotype colorectal cancer. Gastroenterology 2011 Apr;140(4):1174-81. **E7**
48. Kakar S, Aksoy S, Burgart LJ, et al. Mucinous carcinoma of the colon: correlation of loss of mismatch repair enzymes with clinicopathologic features and survival. Mod Pathol 2004 Jun;17(6):696-700. **E8**
49. Kang BW, Kim JG, Lee SJ, et al. Clinical significance of microsatellite instability for stage II or III colorectal cancer following adjuvant therapy with doxifluridine. Med Oncol 2011 Dec;28:Suppl-8. **E11**
50. Kawakami K, Matsunoki A, Kaneko M, et al. Long interspersed nuclear element-1 hypomethylation is a potential biomarker for the prediction of response to oral fluoropyrimidines in microsatellite stable and CpG island methylator phenotype-negative colorectal cancer. Cancer Science 2011 Jan;102(1):166-74. **E7**
51. Kim JC, Roh SA, Cho DH, et al. Chemoresponsiveness associated with canonical molecular changes in colorectal adenocarcinomas. Anticancer Res 2009 Aug;29(8):3115-23. **E10**
52. Kim JC, Choi JS, Roh SA, et al. Promoter methylation of specific genes is associated with the phenotype and progression of colorectal adenocarcinomas. Ann Surg Oncol 2010 Jul;17(7):1767-76. **E7**
53. Kim JE, Hong YS, Ryu MH, et al. Association between deficient mismatch repair system and efficacy to irinotecan-containing chemotherapy in metastatic colon cancer. Cancer Science 2011 Sep;102(9):1706-11. **E10**
54. Kim ST, Lee J, Park SH, et al. Clinical impact of microsatellite instability in colon cancer following adjuvant FOLFOX therapy. Cancer Chemother Pharmacol 2010 Sep;66(4):659-67. **E10**
55. Kim ST, Lee J, Park SH, et al. The effect of DNA mismatch repair (MMR) status on oxaliplatin-based first-line chemotherapy as in recurrent or metastatic colon cancer. Med Oncol 2010 Dec;27(4):1277-85. **E10**
56. Koopman M, Kortman GA, Mekenkamp L, et al. Deficient mismatch repair system in patients with sporadic advanced colorectal cancer. Br J Cancer 2009;100:266-73. **E10**
57. Kweekel DM, Antonini NF, Nortier JW, et al. Explorative study to identify novel candidate genes related to oxaliplatin efficacy and toxicity using a DNA repair array. Br J Cancer 2009 Jul 21;101(2):357-62. **E10**
58. Lamberti C, Lundin S, Bogdanow M, et al. Microsatellite instability did not predict individual survival of unselected patients with colorectal cancer. Int J Colorectal Dis 2007 Feb;22(2):145-52. **E11**
59. Lee DW, Han SW, Lee HJ, et al. Prognostic implication of mucinous histology in colorectal cancer patients treated with adjuvant FOLFOX chemotherapy. Br J Cancer 2013 May 28;108(10):1978-84. **E10**
60. Leopoldo S, Lorena B, Cinzia A, et al. Two subtypes of mucinous adenocarcinoma of the colorectum: clinicopathological and genetic features. Ann Surg Oncol 2008 May;15(5):1429-39. **E10**
61. Li P, Fang YJ, Li F, et al. ERCC1, defective mismatch repair status as predictive biomarkers of survival for stage III colon cancer patients receiving oxaliplatin-based adjuvant chemotherapy. Br J Cancer 2013 Apr 2;108(6):1238-44. **E10**
62. Lievre A, Chapusot C, Bouvier AM, et al. Clinical value of mitochondrial mutations in colorectal cancer. J Clin Oncol 2005 May 20;23(15):3517-25. **E7**
63. Liu X, Zhang H, Lai L, et al. Ribonucleotide reductase small subunit M2 serves as a prognostic biomarker and predicts poor survival of colorectal cancers. Clin Sci 2013 May;124(9):567-78. **E11**
64. Lukish JR, Muro K, DeNobile J, et al. Prognostic significance of DNA replication errors in young patients with colorectal cancer. Ann Surg 1998 Jan;227(1):51-6. **E15**
65. Lv H, Li Q, Qiu W, et al. Genetic polymorphism of XRCC1 correlated with response to oxaliplatin-based chemotherapy in advanced colorectal cancer. Cancer Invest 2013 Jan;31(1):24-8. **E1**
66. Maak M, Simon I, Nitsche U, et al. Independent Validation of a Prognostic Genomic Signature (ColoPrint) for Patients With Stage II Colon Cancer. Ann Surg 2013 Jun;257(6):1053-8. **E2**
67. Mekenkamp LJ, Heesterbeek KJ, Koopman M, et al. Mucinous adenocarcinomas: poor prognosis in metastatic colorectal cancer. Eur J Cancer 2012 Mar;48(4):501-9. **E10**
68. Min BH, Bae JM, Lee EJ, et al. The CpG island methylator phenotype may confer a survival benefit in patients with stage II or III colorectal carcinomas receiving fluoropyrimidine-based adjuvant chemotherapy. BMC Cancer 2011;11:344. **E11**
69. Muller CI, Schulmann K, Reinacher SA, et al. Predictive and prognostic value of microsatellite instability in patients with advanced colorectal cancer treated with a fluoropyrimidine and oxaliplatin containing first-line chemotherapy. A report of the AIO Colorectal Study Group. Int J Colorectal Dis 2008;23:1033-9. **E10**
70. Nakanishi R, Kitao H, Fujinaka Y, et al. FANCJ Expression Predicts the Response to 5-Fluorouracil-Based Chemotherapy in MLH1-Proficient Colorectal Cancer. Ann Surg Oncol 2012 Oct;19(11):3627-35. **E7**
71. Negandhi AA, Hyde A, Dicks E, et al. MTHFR Glu429Ala and ERCC5 His46His polymorphisms are associated with prognosis in colorectal cancer patients: analysis of two independent cohorts from Newfoundland. PLoS ONE [Electronic Resource] 2013;8(4):e61469. **E11**
72. Negri FV, Campanini N, Camisa R, et al. Biological predictive factors in rectal cancer treated with preoperative radiotherapy or radiochemotherapy. Br J Cancer 2008 Jan 15;98(1):143-7. **E6**
73. Nehls O, Okech T, Hsieh CJ, et al. Studies on p53, BAX and Bcl-2 protein expression and microsatellite instability in stage III (UICC) colon cancer treated by adjuvant chemotherapy: major prognostic impact of proapoptotic BAX. Br J Cancer 2007 May 7;96(9):1409-18. **E10**
74. Ogino S, Meyerhardt JA, Irahara N, et al. KRAS mutation in stage III colon cancer and clinical outcome following intergroup trial CALGB 89803. Clin Cancer Res 2009 Dec 1;15(23):7322-9. **E10**
75. Ogino S, Shima K, Meyerhardt JA, et al. Predictive and prognostic roles of BRAF mutation in stage III colon cancer: results from intergroup trial CALGB 89803. Clin Cancer Res 2012 Feb 1;18(3):890-900. **E10**
76. Oh SY, Kim DY, Kim YB, et al. Oncologic Outcomes after Adjuvant Chemotherapy Using FOLFOX in MSI-H Sporadic Stage III Colon Cancer. World J Surg 2013 Jun 11 PMID: 10.1007/s00268-013-2120-8 [doi]. **KQ4E10**
77. Ohrling K, Karlberg M, Edler D, et al. A combined analysis of mismatch repair status and thymidylate synthase expression in stage II and III colon cancer. Clinical Colorectal Cancer 2013 Jun;12(2):128-35. **E13**
78. Park JH, Kim NS, Park JY, et al. MGMT -535G>T polymorphism is associated with prognosis for patients with metastatic colorectal cancer treated with oxaliplatin-based chemotherapy. Journal of Cancer Research & Clinical Oncology 2010 Aug;136(8):1135-42. **E10**
79. Popat S, Zhao D, Chen Z, et al. Relationship between chromosome 18q status and colorectal cancer prognosis: a prospective, blinded analysis of 280 patients.[Erratum appears in Anticancer Res. 2007 Mar-Apr;27(2):1231]. Anticancer Res 2007 Jan;27(1B):627-33. **E7**
80. Rau B, Sturm I, Lage H, et al. Dynamic expression profile of p21WAF1/CIP1 and Ki-67 predicts survival in rectal carcinoma treated with preoperative radiochemotherapy.[Erratum appears in J Clin Oncol. 2004 Feb 1;22(3):576]. J Clin Oncol 2003 Sep 15;21(18):3391-401. **E10**
81. Rego RL, Foster NR, Smyrk TC, et al. Prognostic effect of activated EGFR expression in human colon carcinomas: comparison with EGFR status. Br J Cancer 2010 Jan 5;102(1):165-72. **E11**
82. Ribic CM, Sargent DJ, Moore MJ, et al. Tumor microsatellite-instability status as a predictor of benefit from fluorouracil-based adjuvant chemotherapy for colon cancer. N Engl J Med 2003 Jul 17;349(3):247-57. **E13**
83. Richman SD, Seymour MT, Chambers P, et al. KRAS and BRAF mutations in advanced colorectal cancer are associated with poor prognosis but do not preclude benefit from oxaliplatin or irinotecan: results from the MRC FOCUS trial. J Clin Oncol 2009 Dec 10;27(35):5931-7. **E10**
84. Rijnsoever M, Elsaleh H, Joseph D, et al. CpG island methylator phenotype is an independent predictor of survival benefit from 5-fluorouracil in stage III colorectal cancer. Clin Cancer Res 2003;9:2898-903. **E7**
85. Rosty C, Chazal M, Etienne MC, et al. Determination of microsatellite instability, p53 and K-RAS mutations in hepatic metastases from patients with colorectal cancer: relationship with response to 5-fluorouracil and survival. Int J Cancer 2001 May 20;95(3):162-7. **E10**
86. Roth AD, Tejpar S, Delorenzi M, et al. Prognostic role of KRAS and BRAF in stage II and III resected colon cancer: results of the translational study on the PETACC-3, EORTC 40993, SAKK 60-00 trial. J Clin Oncol 2010 Jan 20;28(3):466-74. **E10**
87. Roth AD, Delorenzi M, Tejpar S, et al. Integrated analysis of molecular and clinical prognostic factors in stage II/III colon cancer. J Natl Cancer Inst 2012 Nov 7;104(21):1635-46. **E10**
88. Sinicrope FA, Rego RL, Halling KC, et al. Prognostic impact of microsatellite instability and DNA ploidy in human colon carcinoma patients. Gastroenterology 2006 Sep;131(3):729-37. **E11**
89. Sinicrope FA, Rego RL, Foster N, et al. Microsatellite instability accounts for tumor site-related differences in clinicopathologic variables and prognosis in human colon cancers. Am J Gastroenterol 2006 Dec;101(12):2818-25. **E11**
90. Sinicrope FA, Rego RL, Halling KC, et al. Thymidylate synthase expression in colon carcinomas with microsatellite instability. Clin Cancer Res 2006 May 1;12(9):2738-44. **E11**
91. Sinicrope FA, Rego RL, Foster NR, et al. Proapoptotic Bad and Bid protein expression predict survival in stages II and III colon cancers. Clin Cancer Res 2008 Jul 1;14(13):4128-33. **E11**
92. Sinicrope FA, Foster NR, Thibodeau SN, et al. DNA mismatch repair status and colon cancer recurrence and survival in clinical trials of 5-fluorouracil-based adjuvant therapy. J Natl Cancer Inst 2011 Jun 8;103(11):863-75. **E13**
93. Sinicrope FA, Foster NR, Yoon HH, et al. Association of obesity with DNA mismatch repair status and clinical outcome in patients with stage II or III colon carcinoma participating in NCCTG and NSABP adjuvant chemotherapy trials. J Clin Oncol 2012 Feb 1;30(4):406-12. **E11**
94. Sinicrope FA, Shi Q. Combining molecular markers with the TNM staging system to improve prognostication in stage II and III colon cancer: are we ready yet? J Natl Cancer Inst 2012 Nov 7;104(21):1616-8. **E5**
95. Smith CG, Fisher D, Claes B, et al. Somatic profiling of the epidermal growth factor receptor pathway in tumours from patients with advanced colorectal cancer, treated with chemotherapy {+/-} cetuximab. Clin Cancer Res 2013 Jun 5. **E10**
96. Sylvester BE, Huo D, Khramtsov A, et al. Molecular analysis of colorectal tumors within a diverse patient cohort at a single institution. Clin Cancer Res 2012 Jan 15;18(2):350-9. **E11**
97. Tang R, Wang JY, Fan CW, et al. p53 is an independent pre-treatment markers for long-term survival in stage II and III colorectal cancers: an analysis of interaction between genetic markers and fluorouracil-based adjuvant therapy. Cancer Lett 2004 Jul 8;210(1):101-9. **E11**
98. Tikidzhieva A, Benner A, Michel S, et al. Microsatellite instability and Beta2-Microglobulin mutations as prognostic markers in colon cancer: results of the FOGT-4 trial. Br J Cancer 2012 Mar 13;106(6):1239-45. **E10**
99. Tran B, Kopetz S, Tie J, et al. Impact of BRAF mutation and microsatellite instability on the pattern of metastatic spread and prognosis in metastatic colorectal cancer. Cancer 2011 Oct 15;117(20):4623-32. **E2**
100. Van Geelen CM, Westra JL, de Vries EG, et al. Prognostic significance of tumor necrosis factor-related apoptosis-inducing ligand and its receptors in adjuvantly treated stage III colon cancer patients. J Clin Oncol 2006 Nov 1;24(31):4998-5004. **E10**
101. Ward RL, Cheong K, Ku SL, et al. Adverse prognostic effect of methylation in colorectal cancer is reversed by microsatellite instability. J Clin Oncol 2003 Oct 15;21(20):3729-36. **E7**
102. Watanabe T, Wu TT, Catalano PJ, et al. Molecular predictors of survival after adjuvant chemotherapy for colon cancer. N Engl J Med 2001 Apr 19;344(16):1196-206. **E10**
103. Westra JL, Schaapveld M, Hollema H, et al. Determination of TP53 mutation is more relevant than microsatellite instability status for the prediction of disease-free survival in adjuvant-treated stage III colon cancer patients. J Clin Oncol 2005 Aug 20;23(24):5635-43. **E10**
104. Wong NA, Morris RG, McCondochie A, et al. Cyclin D1 overexpression in colorectal carcinoma in vivo is dependent on beta-catenin protein dysregulation, but not k-ras mutation. J Pathol 2002 May;197(1):128-35. **E10**
105. Yoon HH, Orrock JM, Foster NR, et al. Prognostic impact of FoxP3+ regulatory T cells in relation to CD8+ T lymphocyte density in human colon carcinomas. PLoS ONE [Electronic Resource] 2012;7(8):e42274.  **E11**
106. Yuanming L, Lineng Z, Baorong S, et al. BRCA1 and ERCC1 mRNA levels are associated with lymph node metastasis in Chinese patients with colorectal cancer. BMC Cancer 2013;13:103. **E1**
107. Zaanan A, Cuilliere-Dartigues P, Guilloux A, et al. Impact of p53 expression and microsatellite instability on stage III colon cancer disease-free survival in patients treated by 5-fluorouracil and leucovorin with or without oxaliplatin. Ann Oncol 2010 Apr;21(4):772-80. **E10**
108. Zaanan A, Flejou JF, Emile JF, et al. Defective mismatch repair status as a prognostic biomarker of disease-free survival in stage III colon cancer patients treated with adjuvant FOLFOX chemotherapy. Clin Cancer Res 2011 Dec 1;17(23):7470-8.**, E10**
109. Zauber NP, Marotta SP, Berman E, et al. Molecular genetic changes associated with colorectal carcinogenesis are not prognostic for tumor regression following preoperative chemoradiation of rectal carcinoma. Int J Radiat Oncol Biol Phys 2009 Jun 1;74(2):472-6. **E11**
110. Zhao DB, Chandler I, Chen ZM, et al. Mismatch repair, minichromosome maintenance complex component 2, cyclin A, and transforming growth factor beta receptor type II as prognostic factors for colorectal cancer: results of a 10-year prospective study using tissue microarray analysis. Chin Med J (Engl) 2011 Feb;124(4):483-90. **E11**
