# Supplementary material for: Systematic review of the predictive effect of MSI status in colorectal cancer patients undergoing 5FU-based chemotherapy
Source: BMC Cancer. 2015 Mar 21;15:156. doi: 10.1186/s12885-015-1093-4 (PMC4376504; doi:10.1186/s12885-015-1093-4)
Supplement: Additional file 2: Figure S1. — Forest plot of hazard ratios (HRs) for the effect of 5FU treatment on disease-free survival (DFS) by MSI status ranked by percentage of stage II patients. By convention, ratios less than 1.0 indicate longer DFS for patients who receive 5FU treatment compared with untreated patients. The test of significance for the difference in the HR for the MSI-H versus MSS groups was not statistically significant (p = 0.111). [file 12885_2015_1093_MOESM2_ESM.doc]

Additional file 2: Forest plot of hazard ratios (HRs) for the eff­ect of 5FU treatment on disease-free survival (DFS) by MSI status ranked by percentage of stage II patients. By convention, ratios less than 1.0 indicate longer DFS for patients who receive 5FU treatment compared with untreated patients. The test of significance for the diff­erence in the HR for the MSI-H versus MSS groups was not statistically significant (p = 0.111).

NOTE: Weights are from random effects analysis

.

.

MSI H

Hong

Sargent

Jover

Hutchins

Kim

Storojeva

Subtotal

(I-squared = 13.9%, p = 0.325)

MSS

Hong

Sargent

Jover

Hutchins

Kim

Storojeva

Subtotal

(I-squared = 7.5%, p = 0.369)

Study

3

5

6

2

5

3

5

6

2

5

Years

Observation

40.4

51.6

58.6

89.3

40.4

51.6

58.6

89.3

% Stage 2

0.42 (0.10, 1.72)

1.53 (0.78, 3.04)

1.02 (0.32, 3.33)

0.81 (0.29, 2.23)

0.49 (0.20, 1.17)

0.49 (0.09, 2.63)

0.84 (0.53, 1.32)

0.50 (0.32, 0.76)

0.70 (0.56, 0.88)

0.49 (0.34, 0.69)

0.59 (0.45, 0.77)

0.67 (0.48, 0.95)

0.81 (0.50, 1.32)

0.62 (0.54, 0.71)

HR (95% CI)

9.36

32.24

13.15

16.92

21.53

6.81

100.00

9.48

31.09

14.02

22.98

14.83

7.60

100.00

% Weight

89

165

60

218

98

21

858

862

436

1695

444

139

N

9.36

32.24

13.15

16.92

21.53

6.81

100.00

9.48

31.09

14.02

22.98

14.83

7.60

100.00

Favors 5FU

Favors No 5FU

1

.5

1

2

NR

NR
