# Supplementary material for: Systematic review of the predictive effect of MSI status in colorectal cancer patients undergoing 5FU-based chemotherapy
Source: BMC Cancer. 2015 Mar 21;15:156. doi: 10.1186/s12885-015-1093-4 (PMC4376504; doi:10.1186/s12885-015-1093-4)
Supplement: Additional file 3: Figure S2. — Forest plot of hazard ratios (HRs) for the effect of 5FU treatment on overall survival (OS) by MSI status ranked by percentage of stage II patients. By convention, ratios less than 1.0 indicate longer OS for patients who receive 5FU treatment compared with untreated patients. The test of significance for the difference in HR for MSI-H versus MSS groups was not statistically significant (p = 0.45). Benatti et al. [28] was included in the figure and point estimate, but this study was removed for calculation of p-values, since it contributed data only to the MSI-H group. [file 12885_2015_1093_MOESM3_ESM.doc]

Additional file 3: Forest plot of hazard ratios (HRs) for the effect of 5FU treatment on overall survival (OS) by MSI status ranked by percentage of stage II patients. By convention, ratios less than 1.0 indicate longer OS for patients who receive 5FU treatment compared with untreated patients. The test of significance for the difference in HR for MSI-H versus MSS groups was not statistically significant (p = 0.45). Benatti et al.^28^ was included in the figure and point estimate, but this study was removed for calculation of p-values, since it contributed data only to the MSI-H group.

NOTE: Weights are from random effects analysis

.

.

MSI-H

Hong

Ohrling

Carethers

Sargent

Benatti

Jover

Barratt

Hutchins

Kim

Storojeva

Subtotal

(I-squared = 0.0%, p = 0.786)

MSS

Hong

Ohrling

Carethers

Sargent

Jover

Barratt

Hutchins

Kim

Storojeva

Subtotal

(I-squared = 63.1%, p = 0.006)

Study

3

10

7

8

5

6

5

10

5

3

10

7

8

6

5

10

5

Years

Observation

40.4

49.6

51.5

51.6

57.7

58.6

60.6

89.3

40.4

49.6

51.5

51.6

58.6

60.6

89.3

% Stage 2

0.57 (0.09, 3.75)

0.85 (0.51, 1.43)

1.16 (0.28, 4.88)

1.56 (0.77, 3.16)

0.55 (0.20, 1.69)

1.08 (0.28, 4.00)

0.94 (0.48, 1.84)

0.71 (0.40, 1.27)

0.61 (0.28, 1.32)

0.49 (0.09, 2.65)

0.85 (0.66, 1.09)

0.34 (0.21, 0.57)

0.86 (0.69, 1.08)

0.66 (0.37, 1.20)

0.74 (0.59, 0.94)

0.46 (0.31, 0.68)

0.73 (0.51, 1.05)

0.86 (0.73, 1.02)

0.59 (0.42, 0.83)

0.84 (0.50, 1.41)

0.68 (0.57, 0.81)

HR (95% CI)

1.89

24.72

3.22

13.18

5.77

3.75

14.55

19.69

10.93

2.30

100.00

7.68

15.04

6.21

14.75

9.88

10.85

16.84

11.42

7.33

100.00

% Weight

89

142

36

165

256

60

89

218

98

21

858

576

168

862

436

279

1695

444

139

N

1.89

24.72

3.22

13.18

5.77

3.75

14.55

19.69

10.93

2.30

100.00

7.68

15.04

6.21

14.75

9.88

10.85

16.84

11.42

7.33

100.00

Favors 5FU

Favors No 5FU

1

.5

1

2

NR

NR
