# Supplementary material for: Systematic review of the predictive effect of MSI status in colorectal cancer patients undergoing 5FU-based chemotherapy
Source: BMC Cancer. 2015 Mar 21;15:156. doi: 10.1186/s12885-015-1093-4 (PMC4376504; doi:10.1186/s12885-015-1093-4)

Supplemental Material: Forest plot of response rate ratio (RR) for the effect of MSI status on response to treatment among patients treated with 5FU therapy. By convention, ratios less than 1.0 indicate a better response to treatment among patients with MSI-L/S status.

Study	Cancer Type	Stage	Test Version	Test Completeness	RR (95% CI)
5FU only					
Ismail 2005	CRC	Advanced	MSI alone	Complete	0.36 (0.13, 1.02)
Brueckl 2003	CRC	Advanced	MSI alone	Complete	0.58 (0.32, 1.07)
Liang 2002	CRC	Advanced	MSI alone	Complete	0.53 (0.38, 0.74)
Rosty 2001	CRC	Advanced	MSI alone	Partial	0.42 (0.17, 1.01)
Charara 2004	Rectal		MSI alone	Complete	(Excluded)
Subtotal (I-squared = 0.0%, p = 0.828)					0.52 (0.40, 0.68)
with estimated predictive interval					. (0.29, 0.93)
.					
5FU+other					
Des Guetz 2007	CRC	Advanced	MSI alone	Complete	1.65 (0.45, 6.11)
Chua 2009	CRC	Advanced	MSI alone	Partial	1.10 (0.27, 4.43)
Muller 2008	CRC	Advanced	MSI+IHC	Complete	2.44 (0.44, 13.45)
Kim 2011	CRC	Advanced	MSI+IHC	Complete	0.81 (0.55, 1.20)
Koopman 2009	CRC	Advanced	MSI+IHC	Complete	1.23 (0.52, 2.90)
Bendardaf 2007	CRC	Mix	IHC alone	Partial	0.20 (0.05, 0.73)
Kim 2010	Colon	Advanced	MSI+IHC	Complete	0.98 (0.65, 1.50)
Subtotal (I-squared = 30.3%, p = 0.197)					0.93 (0.65, 1.33)
with estimated predictive interval					. (0.41, 2.07)
.					
Overall (I-squared = 45.3%, p = 0.051)					0.70 (0.53, 0.94)
with estimated predictive interval					. (0.33, 1.51)

NOTE: Weights are from random effects analysis

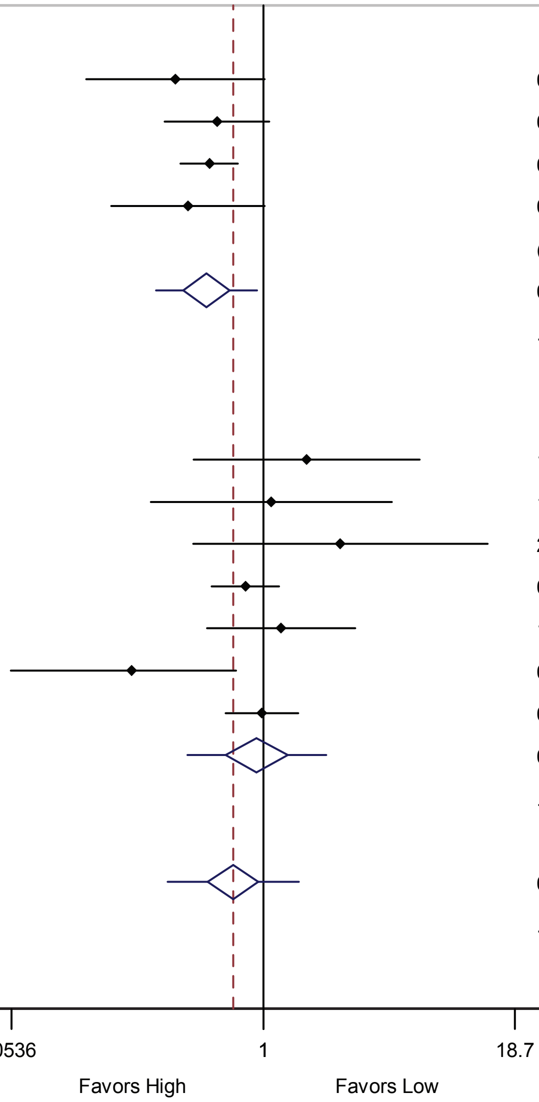

Supplement: Additional file 4: Figure S3. — Forest plot of response rate ratio (RR) for the effect of MSI status on response to treatment among patients treated with 5FU therapy. By convention, ratios less than 1.0 indicate a better response to treatment among patients with MSI-L/S status. [file 12885_2015_1093_MOESM4_ESM.pdf]
